# Use of Social Determinants of Health Z-Codes in Hand Trauma Patients

**DOI:** 10.1177/15589447261437816

**Published:** 2026-05-04

**Authors:** Jessica I. Billig, Michael D. Dang, Jennifer R. Cardin, Joseph H. Joo, Joshua M. Liao

**Affiliations:** 1 University of Texas Southwestern Medical Center, Dallas, USA; 2 Program on Policy Evaluation and Learning, Dallas, TX, USA

**Keywords:** hand, fracture/dislocation, diagnosis, replant, trauma, surgery, specialty, psychosocial, research and health outcomes, health policy, social determinants of health

## Abstract

**Background:**

Hand trauma patients are more likely to be affected by social determinants of health (SDOH) that can adversely affect surgical outcomes. The introduction of z-codes reflecting SDOH has increased the ability to systematically capture SDOH, such as economic circumstances and social support. However, little is known about the uptake of z-codes for identifying SDOH and the characteristics of hand trauma patients with and without documented SDOH z-codes.

**Methods:**

This analysis used 2015-2025 national data from EPIC Cosmos for patients undergoing common surgical procedures for hand trauma. We assessed for the presence of SDOH using z-codes and compared characteristics of patients with and without documented SDOH.

**Results:**

Only 1.7% of surgically treated hand trauma patients had documented SDOH z-codes. Patients with SDOH were younger, Black, living in urban areas, and insured through Medicaid or Medicare compared with patients without SDOH. Between 2015 and 2025, the uptake of z-codes increased from 0.4% in 2015 to 4.1% in 2025, with housing, economic, and social support determinants being most common.

**Conclusions:**

While z-codes enable greater capture of SDOH, adoption was limited over the last decade. Strategies are needed to promote more comprehensive identification of SDOH among surgically treated hand trauma populations.

## Introduction

Social determinants of health (SDOH) such as housing, economic circumstances, or social environment can contribute significantly to clinical, quality, and cost outcomes.^1-5^ While existing research into SDOH in hand surgery patients is limited, studies show that social needs affect patient access to care and patient outcomes. Specifically, studies revealed that language barriers increased patient dissatisfaction with visits leading to shorter visit times and fewer questions asked; underinsured or uninsured status led to worse care coordination; and Medicaid insurance and geographic disparities were associated with delays in access to care.^6-8^

Despite the potential impact of SDOH on patients experiencing hand trauma, it is unknown how adequately SDOH are captured in these populations. This knowledge gap is particularly problematic given that patients with traumatic hand injuries are disproportionately uninsured/underinsured, come from lower socioeconomic backgrounds, and are racially diverse.^9^ In addition, accurately identifying these SDOH is particularly salient in hand trauma patients, as they are more likely to experience traumatic injuries than patients without social needs, resulting in decreased function and increased disability.^10^

Part of the challenge is that in clinical settings, there have not been methods for systematically identifying SDOH. In turn, SDOH screening and documentation have historically been limited. To encourage screening and documentation, International Classification of Diseases, 10th Revision (ICD-10) z-codes were introduced in 2015 to accurately document the socioeconomic and psychosocial needs related to health care utilization.^11,12^ While z-code uptake has been low in other surgical disciplines,^13-15^ little is known about uptake in surgically treated hand trauma patients, or whether patients with documented SDOH z-codes differ from other patients.

## Materials and Methods

### Data Sources and Cohort Creation

**Figure 1.**
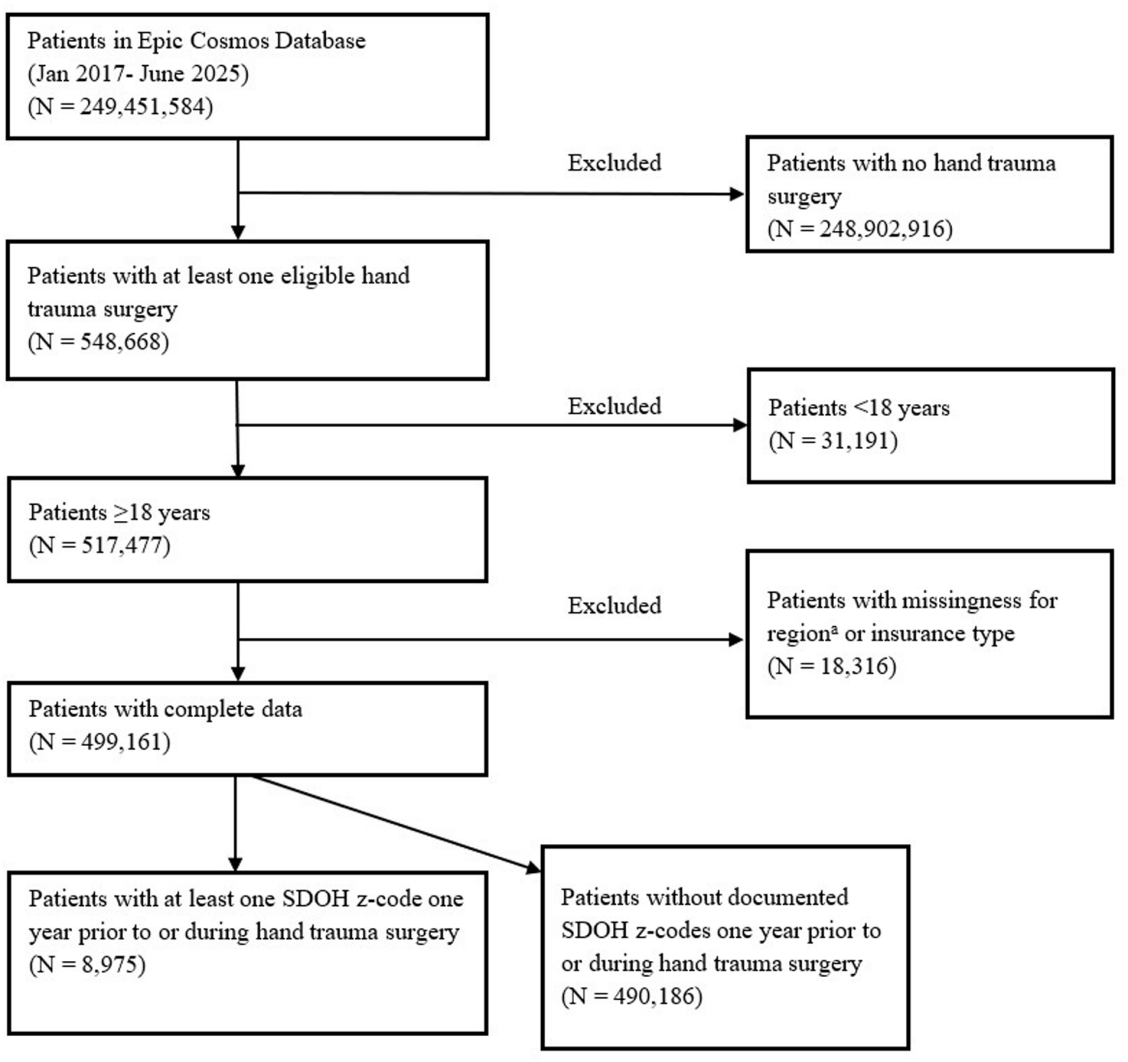
Inclusion and exclusion criteria. *Note.* SDOH = social determinants of health. ^a^Region refers to RUCA, Rural-Urban Commuting Area.

We performed a cross-sectional study in Epic Cosmos, a national database with de-identified information from participating hospitals. Epic Cosmos is an integrated electronic medical record database with 300 million patients from all 50 states within the United States.^16^ Our cohort included patients, aged 18 years or older, undergoing the most common hand trauma-related surgical procedures (derived from Current Procedural Terminology codes, Table 1) from October 2015 until June 2025. Our study period aligns with the introduction of SDOH z-codes into electronic medical record. We included patients with continuous enrollment in EPIC Cosmos starting 1 year prior to their hand trauma surgery to obtain comorbidities. For patients with multiple hand trauma encounters, we included the first encounter. A cohort flow diagram of patient inclusion can be found in Figure 1. Our study was determined to be non-human subjects data given the de-identified nature of study data.

**Table 1.** Current Procedural Terminology Codes for Surgery for Upper Extremity Trauma.

Injury	CPT codes
Distal radius fracture surgical treatment	25606, 2560, 25608, 25609
Flexor tendon repair	26350, 26352, 26356, 26357, 26358, 26370, 26372, 26373
Replantation (finger)	20816, 20822, 20824, 20827
Revascularization (finger/hand)	35206, 35207, 35236, 35266
Metacarpal fracture/dislocation surgical treatment	26607, 26608, 26615, 26742, 26650, 26665, 26676, 26685, 26686, 26706, 26715
Phalangeal fracture surgical treatment	26727, 26735, 26746, 26756, 26765, 26776, 26785
Scaphoid/carpal bone surgical treatment	25628, 25430, 25645, 25431, 25440
Carpal (perilunate)/radiocarpal dislocations treatment	25670, 25695, 25685
Nerve repair	64831, 64832, 64834, 64890, 64910, 64911, 64912, 64913

***Note.*** CPT = Current Procedural Terminology.

### Primary Outcome

Our primary outcome was the presence of 1 or more z-codes in the 1 year prior to, or during, a hand trauma surgery episode (outpatient surgical procedure or inpatient stay associated with hand surgical episode). The ICD-10 z-codes used were Z55-Z65 that document socioeconomic and psychosocial needs of patients (Table 2). Patients were categorized as having versus not having at least 1 documented SDOH z-code.

**Table 2.** Social Determinants of Health ICD-10 Z-Codes.

Z55.X	Problems related to education and literacy
Z56.X	Problems related to employment and unemployment
Z57.X	Occupational exposure to risk factors
Z58.X	Problems related to physical environment
Z59.X	Problems related to housing and economic circumstances
Z60.X	Problems related to social environment
Z62.X	Problems related to upbringing
Z63.X	Other problems related to primary support group, including family circumstances
Z64.X	Problems related to certain psychosocial circumstance
Z65.X	Problems related to other psychosocial circumstances

*Note.* X denotes all subordinate codes within each ICD-10 code category. ICD = International Classification of Diseases, 10th Revision.

### Variables of Interest

We describe characteristics between patients with and without documented SDOH, including age, sex, race, comorbidities, social vulnerability, urban versus residence, and insurance type. In addition to sociodemographic characteristics and comorbidities, we categorized our cohort by procedure type. Procedures were categorized as carpal (perilunate) or radiocarpal dislocations treatment, distal radius fracture surgical treatment, flexor tendon repair, metacarpal fracture or dislocation surgical treatment, nerve repair, phalangeal fracture surgical treatment, finger replantation, hand or finger revascularization, and scaphoid or carpal bone surgical treatment (Table 1). Social vulnerability was measured with The Centers for Disease Control and Prevention and Agency for Toxic Substances and Disease Registry Social Vulnerability Index (SVI).^17^ Social Vulnerability Index as a measure of social vulnerability considers socioeconomic status, household characteristics, racial and ethnic minority status, and housing type and transportation. A higher SVI indicates more social vulnerability; in our analysis, SVI was categorized as low, low-medium, medium high, and high. Urban versus rural residence was measured by Rural-Urban Commuting Area Codes. Insurance was categorized as commercial, Medicaid, Medicare, self-pay, worker’s comp, and other. Comorbidities were determined using the Charlson Comorbidity Index (CCI), a proxy for health status, using ICD-10 codes.^18^ Charlson Comorbidity Index was categorized on a scale from 0 to 4+, with 0 being patients with no comorbid conditions and 4+ being patients with the most comorbidities.

### Statistical Analysis

First, we used descriptive statistics to compare sociodemographic characteristics, comorbidities, and procedure type between patients with versus without documented SDOH. We used χ^2^ tests to compare categorical variables and *t* tests to compare continuous variables. We then looked at z-code utilization over our study period by assessing the number of surgically treated hand trauma patients with documented SDOH z-codes as a proportion of all surgically treated hand trauma patients. We then used χ^2^ test for the utilization of z-code over time. Statistical significance was set at *P* < .001.

## Results

Across our study period, 473 563 patients had a qualifying hand trauma surgery (Table 3). In our cohort, the mean age was 48.6 years, and the majority were female (245, 640; 51.9%) and white (375 711; 79.3%). Most hand trauma patients had a high SVI (142 728; 30.1%), lived in urban areas (391 921; 82.8%), had commercial insurance (167 114; 35.3%), and had few comorbidities (385 082; 81.3% with CCI of 0). The most common procedure was open reduction internal fixation of a distal radius fracture (217 838; 45.9%) (Table 4).

**Table 3.** Characteristics of Hand Trauma Patients With and Without Documented SDOH.

Variables	Total patients (n = 499 161)	Documented SDOH (n = 8975)	No documented SDOH (n = 490 186)	*P* values
Age, mean (SD)	48.5 (18.9)	47.3 (18.3)	48.5 (18.9)	<.001
Sex				
Female, n (%)	257 743 (51.6)	4614 (51.4)	253 129 (51.6)	.7
Male, n (%)	241 418 (48.4)	4361 (48.6)	237 057 (48.4)	
Race				
White, n (%)	394 174 (79.0)	6462 (72.0)	387 712 (79.1)	<.001
Black, n (%)	61 455 (12.3)	1709 (19.0)	59 746 (12.2)	
Asian, n (%)	13 127 (2.6)	146 (1.6)	12 981 (2.6)	
Other,^a^ n (%)	30 405 (6.1)	658 (7.4)	29 747 (6.1)	
SVI				
Low, n (%)	103 084 (20.7)	1229 (13.7)	101 855 (20.8)	<.001
Low-medium, n (%)	118 194 (23.7)	1840 (20.5)	116 354 (23.7)	
Medium-high, n (%)	126 230 (25.3)	2139 (23.8)	124 091 (25.3)	
High, n (%)	151 653 (30.3)	3767 (42.0)	147 886 (30.2)	
RUCA category				
Urban, n (%)	414 389 (83.0)	7646 (85.2)	406 743 (83.0)	<.001
Rural,^b^ n (%)	84 772 (17.0)	1329 (14.8)	83 443 (17.0)	
Insurance				
Commercial, n (%)	175 976 (35.3)	1821 (20.3)	174 155 (35.5)	<.001
Medicaid, n (%)	78 067 (15.6)	2386 (26.6)	75 681 (15.4)	
Medicare, n (%)	111 169 (22.3)	1991 (22.2)	109 178 (22.3)	
Self-pay, n (%)	20 777 (4.2)	440 (4.9)	20 337 (4.2)	
Worker’s compensation, n (%)	14 208 (2.8)	124 (1.4)	14 084 (2.9)	
Other,^c^ n (%)	98 964 (19.8)	2213 (24.6)	96 751 (19.7)	
CCI				
0, n (%)	410 166 (82.2)	6233 (69.4)	403 933 (82.4)	<.001
1, n (%)	44 768 (9.0)	1202 (13.4)	43 566 (8.9)	
2, n (%)	22 053 (4.4)	643 (7.2)	21 410 (4.4)	
3, n (%)	10 048 (2.0)	377 (4.2)	9671 (2.0)	
4+, n (%)	12 126 (2.4)	520 (5.8)	11 606 (2.3)	

*Note.* SDOH = social determinants of health; SVI = Social Vulnerability Index; RUCA = Rural-Urban Commuting Areas; CCI = Charlson Comorbidity Index.

a Other = Alaska Native, Native Hawaiian, or Other Pacific Islander.

b RUCA = Rural combines large rural, small rural, and isolated rural areas.

c Other = Tricare, Third Party Liability, Veterans Affair, other, and otherwise unspecified insurance.

**Table 4.** Hand Trauma Injury in Hand Trauma Patients With and Without Documented SDOH.

Hand trauma injury	Total patients (n = 473 563)	Documented SDOH (n = 8244)	No documented SDOH (n = 465 319)	*P* values
Carpal (perilunate)/radiocarpal dislocations treatment, n (%)	2255 (0.4)	54 (0.6)	2201 (0.4)	<.001
Distal radius fracture surgical treatment, n (%)	228 325 (45.7)	3923 (43.7)	224 402 (45.8)	
Flexor tendon repair, n (%)	29 287 (5.9)	596 (6.6)	28 691 (5.8)	
Metacarpal fracture/dislocation surgical treatment, n (%)	85 017 (17.0)	1737 (19.4)	83 280 (17.0)	
Nerve repair, n (%)	32 275 (6.5)	576 (6.4)	31 699 (6.5)	
Phalangeal fracture surgical treatment, n (%)	92 741 (18.6)	1522 (17.0)	91 219 (18.6)	
Replantation (finger), n (%)	228 (0.1)	6 (0.1)	222 (0.1)	
Revascularization (finger/hand), n (%)	8688 (1.7)	265 (2.9)	8423 (1.7)	
Scaphoid/carpal bone surgical treatment, n (%)	20 345 (4.1)	296 (3.3)	20 049 (4.1)	

*Note.* SDOH = social determinants of health.

Among this sample, 8244 patients (1.7%) had at least 1 documented SDOH. Patients with documented SDOH were on average 1.2 years younger than patients with no documented SDOH (47.3 vs 48.5 years, *P* < .001) (Table 3). There was no statistically significant difference in sex (52.1% female with documented SDOH vs 51.9% female with no documented SDOH; *P* = .6) of patients with or without documented SDOH. Overall, the distribution between the race categories was statistically significantly different between patients with and without documented SDOH (*P* < .001) with a smaller proportion of patients with SDOH being white (65 975; documented SDOH: 72.5% vs no documented SDOH: 79.5%) or Asian (140; documented SDOH: 1.7% vs no documented SDOH: 2.6%). Conversely, a higher proportion of black patients had SDOH (1509; 18.3%) compared with patients with no documented SDOH (55 384; 11.9%). More patients with documented SDOH had high SVI (3430, 41.6%) than patients without documented SDOH (139 298, 29.9%), and the distribution between the SVI categories was statistically significantly different between patients with documented SDOH and no documented SDOH groups (*P* < .001). More patients with documented z-codes lived in urban areas (7100, 86.1%) compared with patients without documented z-codes (384 821, 82.7%), and the difference between rural and urban residency was statistically significant (*P* < .001). There is a statistical difference between type of insurance between patients with and without documented SDOH (*P* < .001), with fewer patients with documented SDOH had commercial insurance (1690, 20.5%) compared with patients without documented SDOH (165 424, 35.6%). More patients with documented SDOH had Medicaid and Medicare insurance (2187, 26.5% and 1872, 22.7%, respectively) than patients without documented SDOH (70 886, 15.4% and 104 845, 22.6%, respectively). The distribution of CCI categories was statistically significant between patients with and without documented SDOH (*P* < .001), with fewer patients with documented SDOH had a CCI of 0 (5538; 67.2%) compared with patients without documented SDOH (379 544; 81.6%) who had a CCI of 0. There was also significant variation in the types of procedures between patients with and without documented SDOH (Table 4).

**Figure 2.**
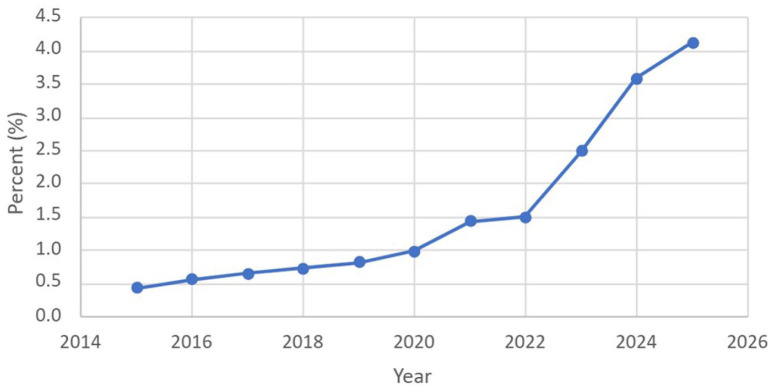
Percentage of hand traumas with social determinants of health z-codes by year, 2015-2025. *Note.* Cochran-Armitage test for trend showed statistical significance with *P* < .001.

The use of z-codes to identify and document SDOH increased over time (*P* < .001). In 2015, 0.4% of patients undergoing surgery for hand trauma had an ICD-10 z-code identifying SDOH compared with 4.1% of patients in 2025 (Figure 2). Throughout the study period, the z-codes that were most often used were Z59—problems related to housing and economic circumstances (79 025 uses)—and Z63—other problems related to primary support group, including family circumstances (35 653 uses). The z-codes that were least used were Z64—problems related to certain psychosocial circumstances (1915 uses)—and Z58—problems related to physical environment (510 uses) (Table 5).

**Table 5.** Counts of SDOH CPT Codes From 2015 to 2025.

Code	Description	Count
Z55	Problems related to education and literacy	7186
Z56	Problems related to employment and unemployment	14 322
Z57	Occupational exposure to risk factors	2025
Z58	Problems related to physical environment	510
Z59	Problems related to housing and economic circumstances	79 025
Z60	Problems related to social environment	17 378
Z62	Problems related to upbringing	6952
Z63	Other problems related to primary support group, including family circumstances	35 653
Z64	Problems related to certain psychosocial circumstances	1915
Z65	Problems related to other psychosocial circumstances	17 286

*Note.* SDOH = social determinants of health; CPT = Current Procedural Terminology.

## Discussion

In this national study, we found that only 1.7% of surgically treated hand trauma patients had SDOH documented through z-codes. Patients who did were more likely to be younger, black, have high SVI, living in urban areas, to be insured through Medicaid or Medicare, and have higher CCI than patients without documented SDOH. The uptake of z-codes increased from 0.4% in 2015 to 4.1% in 2025 with housing, economic, and social support determinants being most common.

This limited uptake of z-codes corroborates prior work. National z-code prevalence has been shown to be approximately 1.4% for fee-for-service Medicare-insured individuals.^19^ In addition, for patients with commercial insurance, only about 0.8% of beneficiaries had a documented z-code, with the majority of encounters recorded in an outpatient setting.^20^ In our study, we find that only 1.7% of hand trauma patients have a documented z-code. However, we would expect higher use of z-codes in our study population given the cohort included individuals with Medicaid, those who are uninsured, and individuals with higher measures of social vulnerability. The adequate measurement of SDOH is needed to determine a comprehensive understanding of the impact of social needs on patient outcomes and provide targeted interventions for at-risk populations.^21^

Considering our findings, prior studies, and evidence that hand trauma patients are more likely to be socially disadvantaged,^9^ there is likely underreporting of SDOH in the hand trauma population. This reflects a need for more comprehensive screening for and documentation of SDOH after hand trauma to improve coordination of patients to necessary resources to address SDOH as well as track health disparities and changes to frequencies of SDOH over time. Barriers to these strategies include a lack of standardization in screening, time, or technological capacities.^21,22^ In addition, providers may feel uncomfortable discussing social needs with patients or struggle to identify solutions to mitigate social needs found on screening.^21,22^ Ways of overcoming these barriers include valuing time spent screening for SDOH with associated financial incentives. This is currently being addressed by the U.S. Centers for Medicare and Medicaid Services with the introduction of Current Procedural Codes code G0136 for billing for SDOH screening. However, it is unknown how these financial incentives will improve the screening for SDOH. Our findings suggest that further interventions are needed to implement standardized screening and documentation protocols and increase provider comfort for screening and documentation so that they may be better equipped to report on and address health inequities.

Our study had limitations. Due to data restrictions, we were unable to ascertain patients’ dual eligibility for both Medicaid and Medicare as well as hospital characteristics. We could not comment on the collection of SDOH information that is not captured by z-codes, but through other screening initiatives. Therefore, we expect that this study underestimates the overall screening of SDOH in the hand trauma population. Finally, the introduction of more SDOH z-codes over time may have contributed to any increase in utilization over time.

Nonetheless, infrequent identification of SDOH using z-codes suggests that additional measures are needed to either encourage z-code use or adopt other avenues for ensuring SDOH screening for surgically treated hand trauma populations.
